# Innovative multi‐scale approach to study the phenotypic variation of seedling leaves in four weedy *Amaranthus* species

**DOI:** 10.1111/plb.13752

**Published:** 2024-12-11

**Authors:** D. Scarpin, G. Este, F. D'Este, F. Boscutti, A. Milani, S. Panozzo, S. Varotto, M. Vuerich, E. Petrussa, E. Braidot

**Affiliations:** ^1^ Department of Agriculture, Food, Environment and Animal Sciences (DI4A) University of Udine Udine Italy; ^2^ Department of Medicine University of Udine Udine Italy; ^3^ NBFC, National Biodiversity Future Center Palermo Italy; ^4^ Institute for Sustainable Plant Protection (IPSP)—National Research Council (CNR) Viale dell'Università 16 Legnaro Italy; ^5^ Department of Agronomy Animal Food Natural Resources and Environment (DAFNAE) University of Padova, Viale dell'Università 16 Legnaro Italy

**Keywords:** Confocal reflection microscopy, imaging, juvenile leaf, leaf traits, multiscale morphometrics, plant phenotyping, weedy amaranths

## Abstract

- Plant phenotyping on morpho‐anatomical traits through image analysis, from microscope images to large‐scale acquisitions through remote sensing, represents a low‐invasive tool providing insight into physiological and structural trait variation, as well as plant–environment interactions. High phenotype diversity in the genus *Amaranthus* includes annual weed species with high invasiveness and impact on important summer crops, and nutritive grain or vegetable crops. Identification of morpho‐anatomical leaf characters at very young stages across weedy amaranths could be useful for better understanding their performance in agroecosystems.
- We used an innovative multi‐scale approach with phenotype analyses of about 20 single‐leaf morphometric traits of four *Amaranthus* species through processing confocal microscopy and camera acquisitions.
- The results highlight that determination of leaf traits at different investigation levels highlight species‐specific traits at a juvenile stage, which are crucial for plant development, competition and establishment. Specifically, leaf circularity and hairiness Aspect Ratio better discriminated *A. tuberculatus* from other species. Also, leaf DW, hairiness area and perimeter variables allowed identification of dioecious amaranth species as distinct from monoecious species.
- The methodology used here provides a promising, reliable and low‐impact approach for the functional characterization of phylogenetically related species and for statistical quantification of traits involved in taxonomy and biodiversity studies.

## Graphical abstract

Multi‐scale phenotyping of young amaranth leaves discriminates different species on the basis of macro‐and microscopic traits.

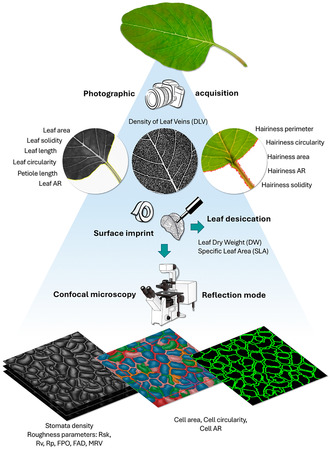

## INTRODUCTION

Plant fitness is determined by the relationship between genotype and environmental factors, leading to phenotype expression, evaluable as different functional types (Pieruschka & Schurr 2019). Gene sequencing technologies have greatly enhanced our knowledge of plant biology (Jiao & Schneeberger 2017) but predicting performance of an individual in each environment cannot be complete if only the gene pool is considered (Pieruschka & Schurr 2019). Therefore, integrated studies of the phenotype are fundamental, considering the enormous variability affecting plant adaptation to different growth conditions (Sultan 2000; Pieruschka & Schurr 2019) and will generate further challenges for modern quantitative analyses (Houle *et al*. 2010). Plants are sessile organisms, permanently bound to the growth site. During evolution plants differentiated many morpho‐anatomical structures necessary to cope with environmental stress and to compete efficiently, allowing these photosynthetic organisms to adapt even to extreme environmental conditions (Nicotra *et al*. 2010). In this context, development of phenotyping science and techniques has emerged as a strategy linking genomics with plant ecophysiology (Li *et al*. 2020). Thanks to the rapid development of imaging technology, computer‐assisted analysis has greatly facilitated scientific research, and provides an important tool for plant phenotyping as a complement or alternative to more limiting and time‐consuming manual measurements (Choudhury *et al*. 2017). High throughput plant phenotyping, in particular, involves analysis of image sequences acquired in different controlled environment conditions (Choudhury *et al*. 2017), allowing acquisition of a large amount of information useful for both holistic (whole plant) and component (single organ) analyses (Das *et al*. 2018).

One phenotyping methodology is the so‐called “Anatomics,” the study and quantification of plant anatomical traits through imaging techniques (Strock *et al*. 2022). This technique allows assessment of variations in leaf vein architecture, hairiness and stomatal density, as well as surface traits related to environmental adaptation of photosynthetic efficiency and water transport (Kattge *et al*. 2020). Leaf trichome structures also vary across individuals and species in shape, size, aspect ratio, and geometric arrangement, and are strongly involved in air–water interactions, hydrophobicity and thermoregulation of the leaf surface (Seale *et al*. 2018; Peters & Noble 2023). Inter‐ and intraspecific variation in morphology and density of leaf trichomes also impact plant performance, since these traits are sometimes implicated in protection from both environmental stresses (e.g. drought, UV damage, extreme temperature) and herbivory attacks or pathogen spore germination (Roy *et al*. 1999; Bickford 2016). In addition, sequestration of toxins and xenobiotics, such as herbicides, are also influenced by both leaf hairiness (Johnson & Baucom 2024) and surface roughness (leaf corrugations and epi‐ and cuticular microstructures) (Wang *et al*. 2015), which ultimately affect leaf wettability (Nairn & Forster 2024). Major vein length per unit area and vein density, together with vein topology and stomatal density contribute to leaf hydraulic conductance and gas exchange, as well as phloem loading, thus affecting photosynthetic carbon assimilation efficiency within and across species (Brodribb *et al*. 2007; Sack & Scoffoni 2013; Pagano *et al*. 2016). At the same time, higher leaf vein density has possible benefits for biomechanical support and protection against herbivores or abiotic stresses (Sack & Scoffoni 2013). High‐throughput anatomical phenotyping is useful in many areas of plant science, from basic research to crop breeding, as plant anatomy is a regulator of several fundamental processes, as well as interactions with other organisms (Lynch *et al*. 2021). Nevertheless, detailed measurement and analysis of anatomical phenotypes is limited, resulting in poor understanding of the extent of phenotypic variation between species and their fitness (Strock *et al*. 2022). New technologies can facilitate both measurement and quantification of anatomical characters, providing more in‐depth information and allow studies even of field‐grown plants (Strock *et al*. 2022).

Phenotyping could represent a further application for more integrated investigations on interactions between crops and weeds, as such analyses could inform development of future target‐directed and less invasive treatments against weeds. Amaranths represent good candidates as plant model for such analysis. The genus *Amaranthus* L. (Amaranthaceae Juss.) contains 70–90 species, mostly annual herbs, found worldwide, covering many different habitats (Milani *et al*. 2020; POWO 2024). The high taxonomic diversity of this genus reflects complex taxonomy and nomenclature issues, many currently unresolved (Bayón 2015; Iamonico 2016, 2020; Iamonico & El Mokni 2018).

The flora of Italy encompasses 22 amaranth species (27 taxa, including subspecies) plus five hybrids. Only two species are considered as native (*A. blitum* L. subsp. *blitum* and *A. graecizans* L. s.l.), others are mostly neophytes native to the Americas (Iamonico 2015a; PFI 2024). *Amaranthus* is one of few dicotyledons with C4 metabolism, allowing it to survive in arid, dry, and high‐salinity environments (Ruth *et al*. 2021) and competing with crops, such as soybean, maize and tomato, due to higher photosynthetic efficiency. The high capacity to hybridize, production of allelopathic substances and high seed production further make many amaranth species extremely invasive (Ward *et al*. 2013), leading to high yield losses among crops, which why some amaranths are considered noxious weeds (Ma *et al*. 2015; Schwartz *et al*. 2016; Milani *et al*. 2020). Reduced weed control through spraying crops is now widespread, making it difficult to manage invasive species (Milani *et al*. 2020) and herbicide‐resistant weed populations have also been reported worldwide (Heap 2024). Dioecious weed species can rapidly evolve resistant biotypes through cross‐fertilization so they show greater genetic recombination and thus high genetic and phenotypic variability (Kreiner *et al*. 2018). Examples of species that have already developed resistance are *A. hybridus* L. and *A. retroflexus* L. (monoecious), *A. palmeri* S. Wats. and *A. tuberculatus* (Moq.) J.D.Sauer (dioecious) (Heap 2024). Resistant biotypes of these four species have been reported in Italy, sometimes all infesting the same field (Milani *et al*. 2020, 2021).

We carried out a phenotyping study on leaf morpho‐anatomical traits of the above *Amaranthus* species, analysing characteristics of the adaxial side of young leaves from individuals grown from seed under the same controlled environment conditions. The decision to analyse these species in early stages of development was determined by the fact that amaranths can become competitive during the crop emergence phase (Massinga *et al*. 2001; Bensch *et al*. 2003), and effective weed control requires foliar application at an early juvenile stage (6–9 true leaf stage, corresponding to BBCH 13–16 (Hess *et al*. 1997)). Second, amaranths can be perfectly controlled with post‐emergence herbicides at early stages of development, whereas treatment of larger plants favours selection for metabolic resistance mechanisms. There are few reports analysing leaf morphometrics in adult crop and weedy amaranth species (Khanam & Oba 2014; El‐Ghamery *et al*. 2017; Terzieva *et al*. 2019; Nyonje *et al*. 2021). Indeed, recent taxonomic identification (Das & Iamonico 2014; Iamonico 2015a; Hassan & Iamonico 2022) and botanical keys (Milani *et al*. 2020) of *Amaranthus* spp. mostly rely on reproductive traits. Besides, plant phenotyping conducted at high resolution of cellular and tissue organization is uncommon compared to whole‐plant organism/ecosystem studies (Schiefelbein 2015; Costa *et al*. 2019). Combined analyses with a top‐down approach have recently increased to address this shortcoming (Amitrano *et al*. 2022). For amaranth, it was expected that evaluation of a considerable number of anatomical and morphological traits of young leaves would help identify at least some *Amaranthus* species. This is particularly relevant in this genus, whose diversity represents a threat and makes it increasingly difficult to eradicate using conventional agronomic procedures using herbicides. Identification of morpho‐anatomical characters expressed ubiquitously in different amaranth species might suggest new treatment protocols to exploit possible weaknesses during the juvenile stages.

## MATERIAL AND METHODS

### Plant Material

Four *Amaranthus* species were considered: *A. hybridus* L., *A. palmeri* S. Wats., *A. retroflexus* L. and *A. tuberculatus* (Moq.) J.D.Sauer. These four are well‐characterized and routinely used for herbicide screenings by the IPSP‐CNR (Institute for Sustainable Plant Protection—National Research Council) research group (Milani *et al*. 2021). *A. hybridus* and *A. retroflexus* were collected from untreated agricultural habitats, while *A. tuberculatus* was collected from the Po riverbank, all in north‐east Italy (Veneto Region). As *A. palmeri* is an alien weed, a wild accession was kindly provided by Prof. T. A. Gaines (Colorado State University) and collected from an untreated agricultural habitat in Georgia, USA (Culpepper *et al*. 2006).

Seeds were sown in 0.6% agar in plastic boxes and placed in a germination chamber at 18°C/28°C night/day and 12‐h photoperiod using neon tubes with a photon flux density of 15–30 μmol m^−2^·s^−1^. The pre‐germinated seedlings were transplanted into plastic pots (11.0 × 10.1 cm) filled with a standard substrate (60% loamy soil, 15% sand, 15% perlite, 10% peat) and grown in a greenhouse (30 °C/20 °C day/night) with a 16‐h light photoperiod (Scarabel *et al*. 2007). Plants were grown until BBCH 13–16 stage at the “Lucio Toniolo” experimental farm in Legnaro (45°21′06.2″N, 11°57′02.9″ E) and immediately transferred to the University of Udine (46°04′52.2″N 13°12′42.7″ E) for image acquisition.

### Image Acquisition

At least 18 plants per species were examined at growth stage 13–16 according to the weed‐extended BBCH scale (Hess *et al*. 1997): the third or fourth leaf from the apex, including the petiole, was collected, and all measurements performed on the same leaf. The image acquisition was conducted at the University of Udine immediately after transferring the plants.

Leaf traits were measured and analysed according to a scale criterion that defines three different groups of morpho‐anatomical traits (see Table 1):

1. Macroscopic traits (full scale leaf analysis),
2. Surface‐related microscopic traits (leaf surface microscopic analysis),
3. Evapotranspiration (ET)‐related traits (full scale and microscopic leaf analysis).

**Table 1:** Definitions of the non‐correlated variables analysed by MANOVA.

leaf analysis level	non‐correlated traits	meaning
Macroscopic traits	Leaf area	Area of the leaf (mm^2^)
	Leaf circularity	Circularity = 4π Leaf area/(Leaf perimeter)^2^ (non‐dimensional)
	Leaf solidity	Area of convex hull that bounds leaf shape as a polygon (mm^2^)
	Leaf length	Leaf major axis (mm)
	Leaf AR	Aspect ratio = leaf major axis/leaf minor axis (non‐dimensional)
	Petiole length	Length of petiole (mm)
	DW	Dry weight (mg)
	SLA	Specific leaf area = leaf area/leaf dry mass (mm^2^/mg)
Surface‐related microscopic traits	Cell area	Area of the cell (μm^2^)
	Cell circularity	Cell circularity = 4 π [cell area/(cell perimeter)^2^] (non‐dimensional)
	Cell AR	Aspect ratio = cell major axis/cell minor axis (non‐dimensional)
	Rsk	Skewness of the assessed epidermis profile
	Rv	Lowest valley
	Rp	Highest peak height
	FPO	Average polar facet orientation (between 0 and 90 sexagesimal degrees)
	FAD	Average direction of azimuthal facets (between 0 and 360 sexagesimal degrees)
	MRV	Vector resulting from average of inclinations of all cellular facets
ET‐related traits	Hairiness area	Tomentosity area (mm^2^)
	Hairiness perimeter	Tomentosity perimeter (mm)
	Hairiness AR	Aspect ratio of hairs = hair major axis/hair minor axis (non‐dimensional)
	Hairiness circularity	Hair cell circularity = 4 π [hair area/(hair perimeter)^2^]
	Hairiness solidity	Area of convex hull bounds hair shape as a polygon (mm^2^)
	DLV	Density of Leaf veins = total vein length/area (mm/mm^2^ or %) (Price *et al*. 2011)
	Stomata density	(Stomata/mm^2^)

ET, Evapotranspiration.

Leaves were placed on a white light LED panel (RaLeno Photographic Equipment, Shenzhen, China) for full‐scale image acquisition, with the adaxial side facing upwards. A glass sheet was used to keep them flat (Figure S1). Photos were taken with a Panasonic DC‐GH5 camera (Panasonic, Osaka, Japan) at a 60‐mm focal length (settings: f/22, 1/800 s, ISO 800), at a known constant distance from the leaf.

Leaf surface imprints (Sun *et al*. 2019; Zajicova *et al*. 2019) were prepared to examine the morphological parameters of epidermal cells, number of stomata and roughness‐related traits of the epidermal tissue (see Table 1): the median portion of the adaxial leaf side was coated with a double layer of transparent nail polish, allowing the first coat to dry before applying the second. After complete drying, the resulting imprint was removed using adhesive tape and tweezers and mounted in distilled water on a microscope slide, under a # 1 coverslip sealed with the same polish.

Samples were imaged in reflection mode on a Leica TCS SP8 confocal microscope (Leica Microsystems, Wetzlar, Germany) using a 40×/1.10 NA water immersion objective and a 488 nm laser line. *Z*‐stacks covering reflection from the whole surface of the field were collected at 1 AU pinhole aperture and 0.422 μm step size. Maximum intensity projection images (Figure S2) were generated using Leica Application Suite X (LAS X) v 3.5.5 software. Alternatively, the z‐series were processed for roughness‐related trait analysis as described below.

### Image Analysis

**Fig. 1:**
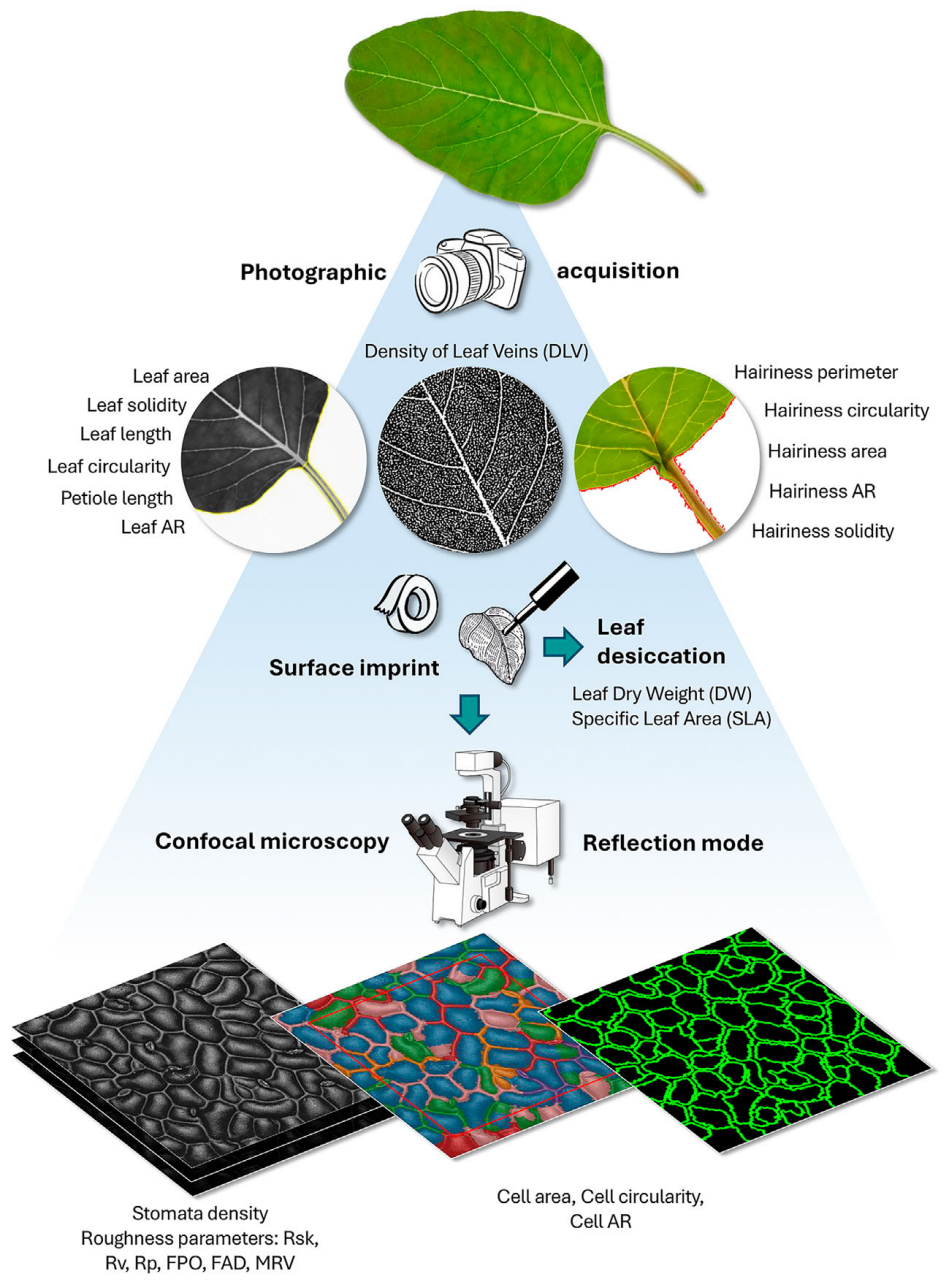
Description of traits analysed from macroscopic (full‐scale) to a microscopic level. See Table 1 for abbreviations.

The images were analysed using *Fiji* software (*win‐64* version) (Schindelin *et al*. 2012) to measure the different morpho‐anatomical parameters. The entire workflow is presented in Fig. 1, and all morpho‐anatomical traits examined are reported in Table 1.

#### Macroscopic traits

Both the software default tools and the *LeafJ* plugin (Maloof *et al*. 2013) were used to analyse morpho‐anatomical macroscopic leaf traits. The following parameters were measured: leaf area, circularity (a leaf shape descriptor representing the ratio between leaf area and true perimeter or, more precisely: 4 π [leaf area/(leaf perimeter)^2^]), length (maximum length in mm), aspect ratio (AR, leaf major axis/leaf minor axis), solidity (area of the convex hull that bounds the leaf shape as a polygon) and petiole length (mm) (see Table 1).

After image acquisition was completed, leaves were placed overnight in an oven at 70°C then weighed to determine Dry Weight (DW). This parameter, together with the previously measured leaf area, was used to calculate Specific Leaf Area (SLA) (Kutbay *et al*. 2016; Riva *et al*. 2016).

#### Surface‐related microscopic traits

Confocal maximum intensity projections were used to analyse the morpho‐anatomical parameters of tegument cells; in this case, analysis was conducted by combining the *Fiji* software with that of *LeafNet* website (Li *et al*. 2022). Specifically, *LeafNet* was used to segment tegument cells, while cell counting and measurement of cell area, circularity, and Aspect Ratio (AR) (see Table 1) were performed with *Fiji*. Finally, a series of parameters concerning roughness‐related traits were measured from the confocal reflection z‐stacks of the nail polish imprint, according to the McNaughtion protocol (McNaughtion 2012). First, data were processed with the *Extended Depth of Field Fiji* plugin (Forster *et al*. 2004), followed by analysis of the resulting height map with *SurfCharJ_1q* plugin (Chinga *et al*. 2007) to calculate Rsk, Rv, Rp, FPO, FAD, MRV values (see Table 1) (Chinga *et al*. 2007). For a more detailed graphical description of the traits, it is recommended to consult a specific website (KEYENCE International 2024).

#### Evapotranspiration (ET)‐related traits

The *Vessel Analysis* plugin (Vessel Analysis 2023) was used to measure Density of Leaf Veins (DLV) as a percentage (Price *et al*. 2011) directly on full‐scale leaf images, while *Fiji* thresholding tools were applied to measure hairiness area, perimeter, circularity, Aspect Ratio (AR) and solidity (see Table 1). Moreover, a manual counting was performed for stomata density using maximum intensity projections of the confocal reflection z‐stacks; the obtained value was then transformed into the number of stomata per leaf area.

### Statistical Analysis

A Pearson correlation test was first applied to test for variable collinearity. In the case of two or more highly correlated variables (r > |0.75|), only one variable from the group was considered (Dormann *et al*. 2013).

A Multivariate Analysis of Variance (MANOVA) was carried out using the *R Studio* software (2022.02.0–443 version) to test trait differences between the four study species. Where significant (*P* < 0.05), the MANOVA models were used to perform a Canonical Discriminant Analysis (CDA). For each CDA analysis, a Likelihood Ratio test was performed to verify model accuracy (results not shown). MANOVA and CDA were performed on each trait group, separately (leaf macroscopic, surface‐ and ET‐related traits).

Following outcomes of the CDAs, variables with a high score and, therefore, having a significant effect in the statistical model were chosen for further univariate analyses. For all variables (see Table 3) a Shapiro–Wilk normality test was performed to determine normal distribution of the data. In the case of variables without a normal distribution, transformation was performed by conversion with appropriate functions (Table 3). An ANOVA was then performed to test for differences between the study species for each selected trait (*P* < 0.05). Where significant, a *post‐hoc* analysis using the LSD Fisher test was used to check pairwise comparisons between species.

## RESULTS

Several traits of the amaranth leaf were measured at different scales, starting from macroscopic (full‐scale), down to a microscopic level. This approach was used to build a phenotyping model to describe as accurately as possible the variability of morpho‐anatomical traits in the four amaranth species studied.

### MANOVA Analysis

**Fig. 2:**
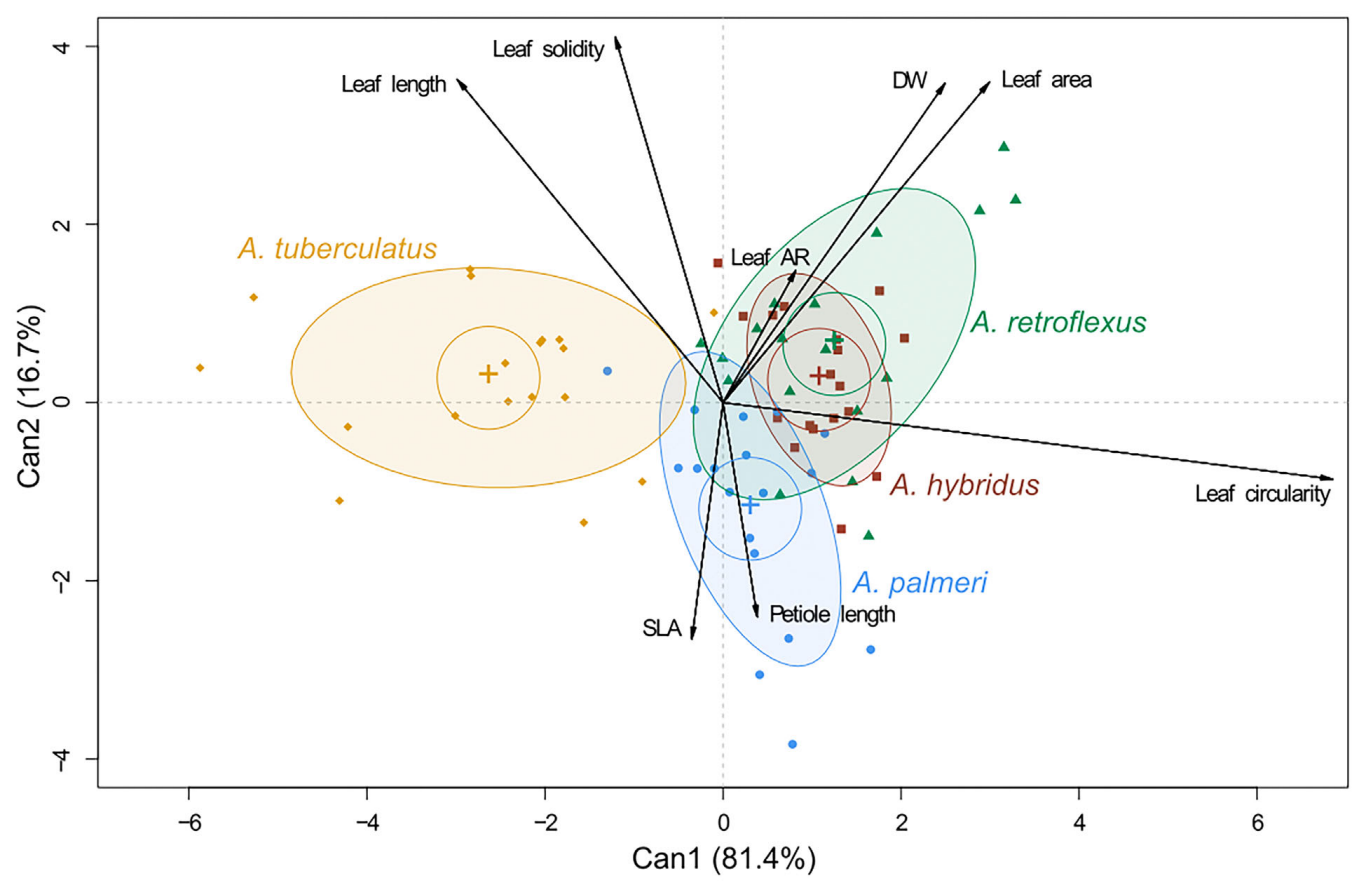
Canonical Discriminant Analysis (CDA) biplot of morpho‐anatomical leaf traits analysed at macroscopic scale. Can1 and Can2, two first canonical variables; DW, dry weight; SLA = specific leaf area.

**Fig. 3:**
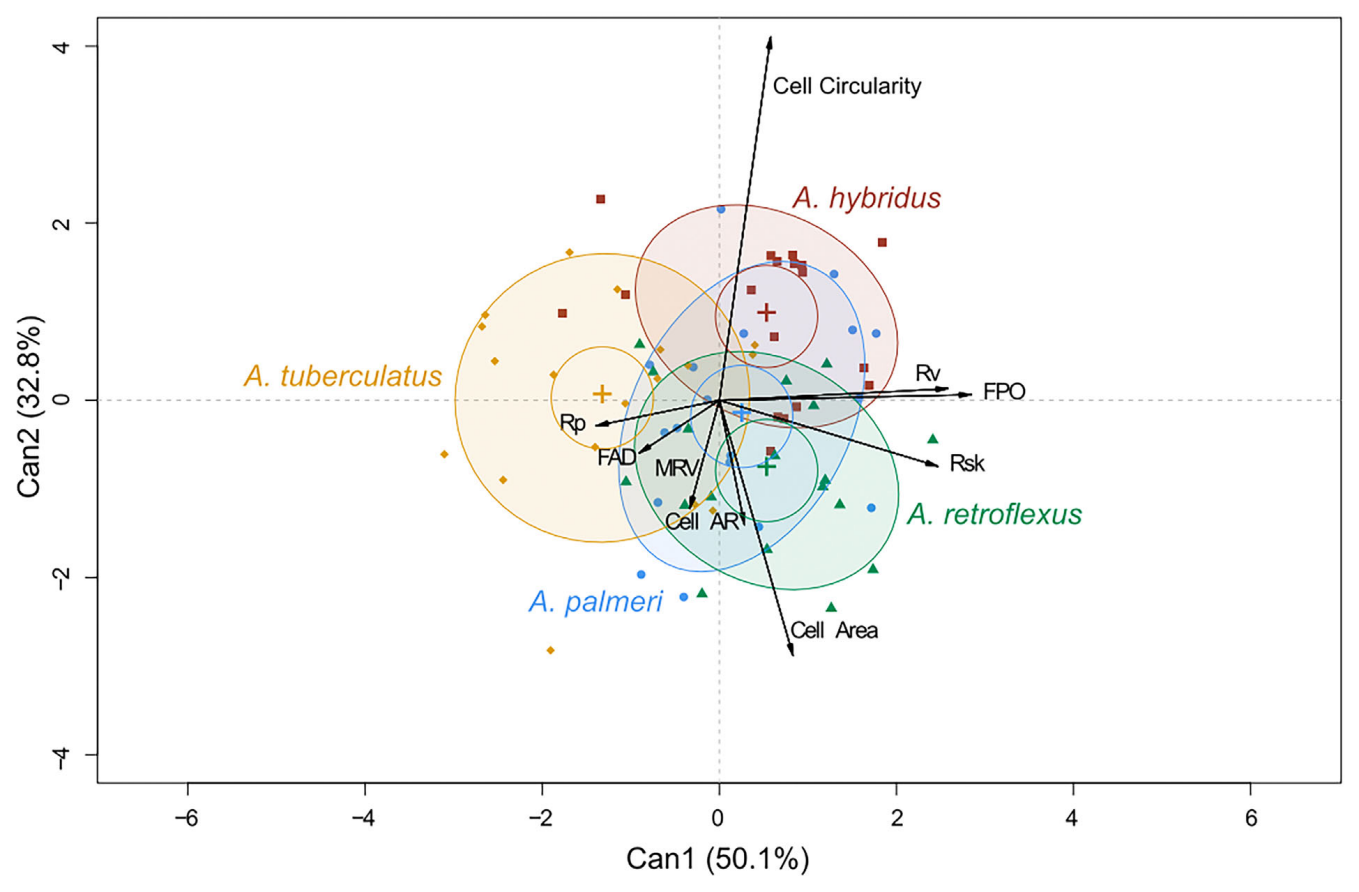
CDA biplot of morpho‐anatomical traits of leaf surface analysed at microscopic scale. Can1 and Can2, two first canonical variables; Rp, highest peak height of surface; FAD, average direction of azimuthal facets; MRV, vector resulting from average of inclinations of all cellular facets; Cell AR, cell aspect ratio; Rv, lowest valley of surface; FPO, average polar facet orientation; Rsk, skewness of assessed epidermis profile.

**Fig. 4:**
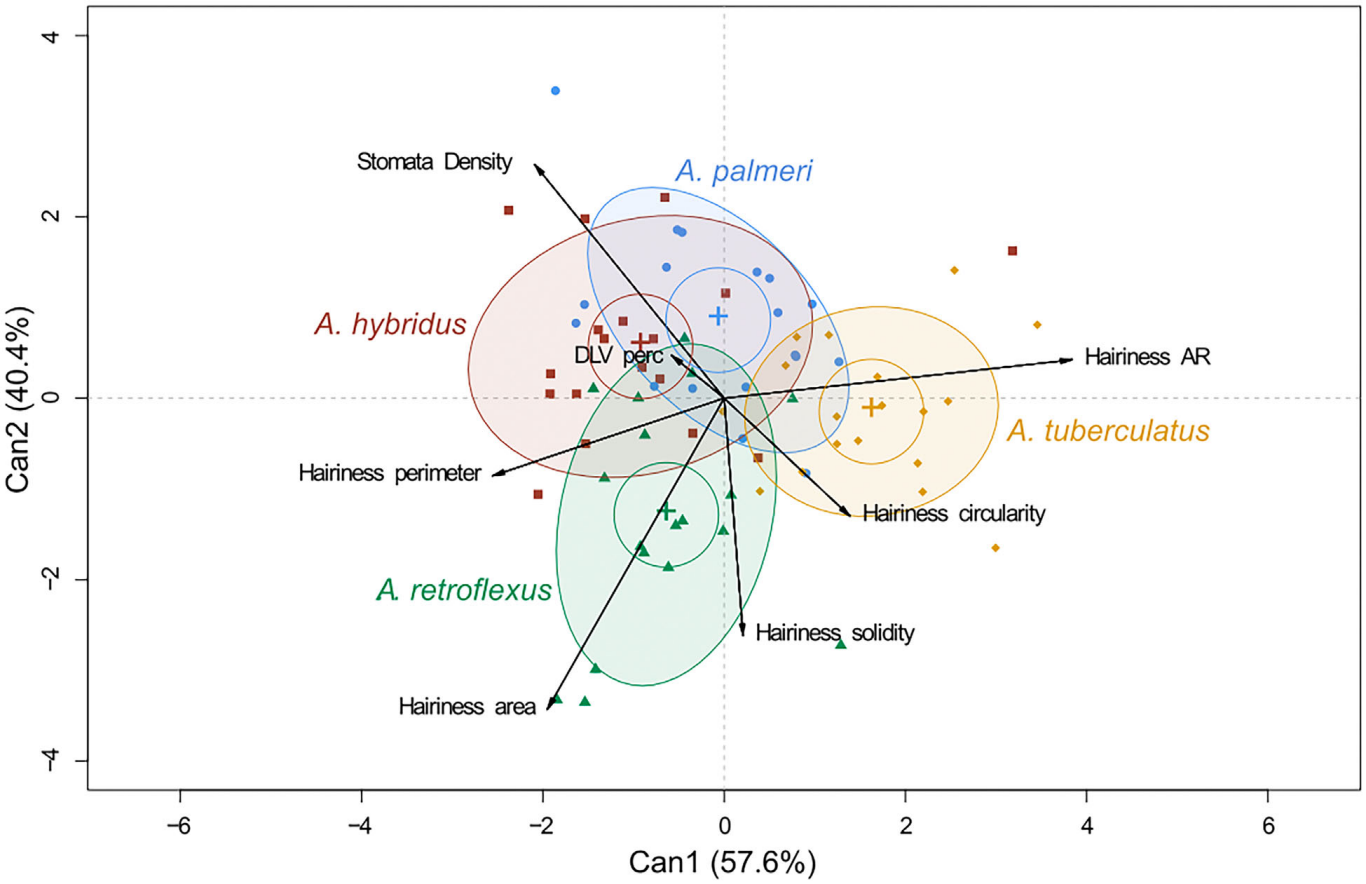
CDA biplot of morpho‐anatomical leaf traits related to evapotranspiration (ET) analysed at microscopic scale. Can1 and Can2, two first canonical variables; Hairiness AR, hairiness aspect ratio; DLV perc, density of leaf veins percentage.

The analysed variables (Table 1) were grouped into three different sets according to morpho‐anatomical role and subjected to MANOVA to highlight not only the percentage variance explained by the model, but also correlations between the factor ‘species’ and the different traits measured. The multivariate analysis model (Table 2) had high significance for all three considered areas. These conclusions were confirmed by subsequent Canonical Discriminant Analysis (CDA) (Figs 2, 3, 4).

**Table 2:** MANOVA of morpho‐anatomical leaf traits at different scale levels.

Leaf analysis level	MANOVA	Df	pillai	approx. *F*	Num–Den Df	*P*	significance
Macroscopic traits	(Intercept)	1	0.999	69697.0	8–61	<0.001	***
	Species	3	1.125	5.0	24–189	<0.001	***
	Residuals	68					
Surface‐related microscopic traits	(Intercept)	1	0.998	5361.3	9–60	<0.001	***
	Species	3	0.855	2.7	27–186	<0.001	***
	Residuals	68					
ET‐related traits	(Intercept)	1	0.996	2512.4	7–62	<0.001	***
	Species	3	0.963	4.3	21–192	<0.001	***
	Residuals	68					

Df, degrees of freedom; ET, Evapotranspiration; F, Fisher; Num‐Den Df, numerator—denominator of Df; *P*, *P* value; Pillai, Pillai value.

*** *P* < 0.001.

To simplify analyses of the different studied traits, a subdivision according to scale of the analysis or of the regulatory function of leaf gas exchange was adopted. Fig. 2 describes the CDA on leaf traits measured at macroscopic level. Canonical variable 1 (Can1) explained a significant percentage of the variance (81.4%), which exceeds 98% if Can2 (16.7%) is also considered. The CDA showed that data for *A. tuberculatus* are in the negative portion of the biplot and are clearly distinguishable from the other three ellipses related to *A. hybridus*, *A. retroflexus* and *A. palmeri*. Furthermore, the distribution of vectors associated with different traits shows that these species share a positive correlation with the trait ‘leaf circularity’ and to some extent ‘DW’ and ‘leaf area’, and a negative correlation with ‘leaf length’, as evidenced by the DIM plot associated with the CDA (Figure S3). The graph shows that *A. tuberculatus* had an opposite trend.

The CDA applied to variables comprising traits related to leaf surface measured at microscopic scale (Fig. 3) shows a more homogeneous distribution between the two canonical variables of the explained variance, which together reached 82.9%. Again, *A. tuberculatus* was clearly distinct from the other three species, whose ellipses were in the positive portion of the biplot and show a high positive correlation with traits in this range, especially with some roughness‐related variables such as Rsk, Rv and FPO, but less so for cellular parameters such as ‘area’, ‘circularity’ and ‘AR’ (Fig. 3). The only Rp character is positively correlated with *A. tuberculatus*, which again shows mirrored layout compared to the other three species. The negative score attributed to this in the DIM plot (Figure S4) is therefore attributable to correlation with the traits negatively correlated with ‘Can1’ (Rp and FAD) and further to the inverse correlation with ‘Rsk’, ‘Rv’ and ‘FPO’, variables that positively influence the score. Nevertheless, absolute values of the scores showed little variation, confirming that the analysis in this second area returned a Can1 parameter capable of explaining only 50% of the variability.

The CDA analysis of traits involved in leaf evapotranspiration (Fig. 4, Figure S5) explained by the two main canonical variables was collectively 98% while the percentage difference between Can1 and Can2 was lower (57.6% and 40.4%, respectively). Again *A. tuberculatus* differs from the other species in traits involved in leaf conductivity (Fig. 4). In fact, the ellipsis relative to *A. tuberculatus* is in the right portion of the biplot, whereas the other species (*A. hybridus*, *A. palmeri* and *A. retroflexus*) are placed in the left‐hand side. Can1 can be related to a gradient of hairiness shape, mainly linked to the variables ‘hairiness AR’, ‘hairiness circularity’ and inversely with ‘hairiness perimeter’ (Figure S5).

The variance explained by Can2 demonstrated that *A. retroflexus* is clearly separated from *A. hybridus*, *A. palmeri* and *A. tuberculatus* by changes in the traits ‘hairiness area’ and ‘hairiness solidity’ (mainly contributing to Can2) and the vector ‘stomata density’, which also contributed to Can2, but with an opposite trend. Accordingly, the DIM plot with the box‐plot analysis related to Can 1 (Figure S5) confirms that *A. tuberculatus* had a positive score related to the high positive correlation with the ‘hairiness AR’ and the partial correlation with the ‘hairiness circularity’, while *A. palmeri* (also a dioecious species) had a value slightly above zero. In contrast, the two monoecious species (*A. hybridus* and *A. retroflexus*) are characterized by negative scores that can be explained by the high correlation with the negative vectors ‘hairiness perimeter’, ‘hairiness area’ and ‘stomata density’.

The results of the CDA highlight which traits are significant in discriminating between different amaranth species and which were also highly correlated with the multivariate model and, therefore, able to adequately explain the variability observed.

### ANOVA Evidencing Most Effective Traits

As confirmation of the CDA, the traits were individually subjected to one‐way ANOVA (Table 3). All considered variables showed a high degree of significance in the ANOVA (at least *P* < 0.01), and in some cases, also revealed significant differences when the LSD *post‐hoc* test was applied. Among the macroscopic traits, leaf area was highly significant (Table 3) due to higher averages in the two monoecious species: only *A. retroflexus* and *A. tuberculatus* were significantly different for this trait (Figure S6). Likewise, for the leaf DW trait, *post‐hoc* analysis discriminated amaranth species on a taxonomic basis, since it separated monoecious from dioecious species (Figure S7). The leaf circularity variable significantly differed among species, where only the mean of *A. tuberculatus* was significantly lower after the LSD test when compared to the others (Figure S8). On the contrary, leaf length of *A. tuberculatus* differed significantly only from that of *A. palmeri* (Figure S9).

**Table 3:** ANOVA of morpho‐anatomical leaf traits at different scales.

leaf analysis level	trait		Df	sum sq	*F* value	*P*	significance
Macroscopic traits	Leaf area	Transformation: ln (*Leaf area*)					
		Species	3	1.22	4.988	0.003	**
		Residuals	68	5.54			
	Leaf circularity	Transformation: (*Leaf circularity*)^3^					
		Species	3	0.47	35.644	<0.001	***
		Residuals	68	0.30			
	Leaf length	Transformation: none					
		Species	3	298.61	4.882	0.004	**
		Residuals	68	1386.43			
	Leaf dry weight	Transformation: none					
		Species	3	2.36	5.094	0.003	**
		Residuals	68	10.52			
Surface‐related microscopic traits	FPO	Transformation: (*FPO*)^5^					
		Species	3	3.64 e^+18^	4.852	0.004	**
		Residuals	68	1.70 e^+19^			
	Cell area	Transformation: sqrt (*Cell area*)					
		Species	3	392.55	4.456	0.006	**
		Residuals	68	1996.81			
	Cell circularity	Transformation: none					
		Species	3	0.11	6.813	<0.001	***
		Residuals	68	0.37			
ET‐related traits	Hairiness AR	Transformation: *1/Hairiness AR*					
		Species	3	0.26	11.929	<0.001	***
		Residuals	68	0.49			
	Hairiness area	Transformation: *log (hairiness area)*					
		Species	3	2.04	7.784	<0.001	***
		Residuals	68	5.95			
	Hairiness perimeter	Transformation: none					
		Species	3	15941	4.540	0.006	**
		Residuals	68	79586			
	Stomata density	Transformation: sqrt (*Stomata density*)					
		Species	3	114254	5.686	0.002	**
		Residuals	68	455456			

Df, degrees of freedom; ET, evapotranspiration; F, Fisher, *P, P* value; Sum Sq, sum of squares.

** *P* < 0.01.

*** *P* < 0.001.

Regarding microscope characters related to roughness, the ANOVA gave a more differentiated pattern. On the one hand, the variable FPO again was significantly different between *A. palmeri* and *A. tuberculatus* for the LSD test (Figure S10), which causes different leaf surface behaviour when studying the Bi‐directional Scattering Distribution Function (Comar *et al*. 2012). On the other hand, the tegument cell area was significantly different in the LSD test among the two monoecious species because the epidermal cell area of *A. retroflexus* was about one‐third larger than that of *A. hybridus* (Figure S11).

Cell circularity also differed in the *Amaranthus* species studied. The shape of cells of *A. hybridus* most closely approximated a circumference and differentiated them significantly from those of *A. palmeri* and *A. retroflexus* for this trait (Figure S12).

The last variables considered include traits that modulate gas exchange between leaf and environment and, in accordance with the CDA, the significance of the species as an independent variable was demonstrated by ANOVA. In particular, hairiness AR of hairs distributed along the leaf perimeter had a significant higher ratio between major and minor axes in *A. tuberculatus* (Figure S13), compared to the other three species. This variable could be considered a reliable trait that supports phylogenetic analysis for *A. tuberculatus* identification. Stomata density of *A. tuberculatus* (Figure S16) was lowest of the four species; however it was significantly different only from that of *A. hybridus*, but in this case it was not clear evidence for taxonomic discrimination among the four amaranths. Conversely, both hairiness area and hairiness perimeter (Figures S14, S15) discriminate significantly between monoecious species, known to be characterized by a tomentose pattern, with respect to the glabrous dioecious weeds, *A. tuberculatus* and *A. palmeri*.

## DISCUSSION

In recent years, non‐invasive imaging techniques have been developed for quantitative studies of plant traits related to growth and adaptation to biotic and abiotic stress, extending from microscope acquisitions to high‐scale imaging through remote sensing (Li *et al*. 2014, 2020). Here we used an integrated multiscale approach (whole leaf, tissue and cells) on single leaf phenotypic variation among four related *Amaranthus* summer weeds (*A. hybridus*, *A. retroflexus A. palmeri*, and *A. tuberculatus*). These species commonly invade soybean fields of northern Italy, becoming more and more noxious because some populations evolve cross‐resistance to the most commonly used herbicide mode of action for their control: acetolactate synthase (ALS) inhibitors (Milani *et al*. 2020, 2021; Gruppo Italiano Resistenza Erbicidi (GIRE) 2024).

We combined conventional digital leaf morphometrics with further analysis by scaling to a higher spatial resolution, through processing of reflection confocal microscopy acquisitions on adaxial leaf surface imprints. This last technique avoids time‐consuming histochemical sample preparation and allows visualization of the leaf surface and cell structure of the epidermis, thus providing quantitative anatomical traits essential to functional processes of the leaf/plant, such as photosynthesis, hydraulic conductance, plastic acclimation, and adaptation to environmental changes (Amitrano *et al*. 2022; Strock *et al*. 2022). In this respect, many authors advocate the need to address the lack of integration between high‐throughput, whole plant phenotyping analysis and quantification of in‐depth anatomical or molecular traits of leaves at different scales of organization (Granier & Tardieu 2009; van Eeuwijk *et al*. 2019; Amitrano *et al*. 2022). This represents one of the major challenges in the plant phenotyping approach, as a technology useful for implementation and transition to precision and digital agriculture (Costa *et al*. 2019) and development of more resilient agroecosystems (Janni & Pieruschka 2022).

In addition to a synchronization in analysis of different species at a very similar early growth stage, another challenge was assessing multi‐scale phenotyping analysis on younger, immature leaf blades of weeds, when, for example, leaf hairs are not completely differentiated (Telfer *et al*. 1997). The multi‐scale phenotyping analysis on young leaves proved to be accurate, reproducible, reliable, and capable of discriminating across the four studied amaranth species. In addition to quantitative (continuous) parameters, our analysis included automated assessment of qualitative (categorical) variables, which commonly involve visual inspection in traditional manual phenotyping and do not include statistical analysis because of difficulties in quantification. More specifically, CDA analysis showed that variables comprising leaf macroscopic characters (leaf circularity and leaf DW) and hairiness traits (hairiness area, hairiness perimeter and hairiness Aspect Ratio—AR) were the most important in explaining the high variance related to taxonomic distance. In both leaf circularity and hairiness AR this effect distinguished *A. tuberculatus* from the other three species. Some results were expected, given the elongated, lance‐shaped leaves in this first species, noticeable also at the first stages of development (Figure S1). Also, our results confirmed that the relationships between leaf shape and size traits are strongly correlated (Wäldchen & Mäder 2018) and that taxonomy is one of the main drivers of leaf shape variation at the juvenile stage. We found that *A. tuberculatus* had more distinct macroscopic morpho‐anatomical characters and, therefore, may have followed a separate evolutionary path. Supporting this statement, recent studies on phylogenetic relationships and size of the genome in the genus *Amaranthus* confirm that the monoecious *A. retroflexus* and *A. hybridus* belong to the same subgenus *Amaranthus*, while the two dioecious species, *A. palmeri* and *A. tuberculatus*, although included in the same subgenus *Acnida* (L.) Aellen ex K.R. Robertson have been described as phylogenetically divergent (Wassom & Tranel 2005; Stetter & Schmid 2017). However, based on Waselkov *et al*. (2018), the infrageneric classification of Mosyakin & Robertson (1996) is not natural, since it does not match the clades as identified in phylogenetic trees; this evidence was later highlighted in morphometric studies (Iamonico *et al*. 2023).

Leaf phenotyping variation associated to leaf shape and AR is tuned to optimize photosynthetic capacity and growth, by influencing light absorption and gas exchange (Chitwood *et al*. 2014; Zhang *et al*. 2023). In particular, *A. tuberculatus* has smaller leaf circularity values, which are linked to serrations and lobes and to elongated shape (Li *et al*. 2018). This morphometric parameter, as well as AR and solidity, have previously been used to measure the genetic basis of shape variation in several crop species, being strongly related to important yield traits (Chitwood *et al*. 2014, 2015; Gupta *et al*. 2020; Rowland *et al*. 2020). Interestingly, some of the measured characters, both macroscopic (leaf area and leaf length) and microscopic (cell area, cell circularity, FPO, ET, stomata density) are less efficient markers for a taxonomic classification, since they partially contrast with the current classification models (Wassom & Tranel 2005; Stetter & Schmid 2017; Raiyemo & Tranel 2023). However, the high measured variability demonstrated here that these traits could be reliable indicators for amaranth variability.

Among morphometrical traits explaining leaf variation *vs*. evapotranspiration, hairiness AR of marginal trichomes was an additional parameter useful in distinguishing *A. tuberculatus* from the other three species. This species has significantly high hairiness AR (inversely correlated to hairiness roundness) compared to the other amaranth species. The structure of leaf surface is influenced at three different levels—trichome shape and density and protruding veins– by cell size shape and undulation, and by shape and size of the epicuticular wax system (Boize *et al*. 1976; Wang *et al*. 2015). All these traits affect plant health and adaptation to environmental stresses (Garcia *et al*. 2022; Peters & Noble 2023), and pesticide wetting (Johnson & Baucom 2024) by affecting herbicide distribution and absorption. However, other variables of leaf tegument structure at this stage did not discriminate across specific leaf phenotypes, apart from stomata density, for which *A. tuberculatus* differs significantly from *A. hybridus*. This could be because juvenile leaf epidermal cells generally do not produce epidermal hairs, except at the leaf margins or tip (Bongard‐Pierce *et al*. 1996), so that trichome differentiation on the adaxial surface was not still completed at the time of image acquisition. Hence, we cannot rule out that at older stages the hairiness trait module could better explain effects of genotype or environment on leaf phenotypic diversity. Such evidence confirmed the empirical observation that dioecious species, such as *A. tuberculatus* and *A. palmeri*, are mostly glabrous (Iamonico 2015b; Vélez‐Gavilán 2019), given the inverse correlation with hairiness area and perimeter, when analysed by CDA. Conversely, *A. hybridus* and A. *retroflexus* are monoecious and exhibited a positive correlation with the same traits, according to a more tomentose aspect (Khan 2021; Shehzadi *et al*. 2022). Trichomes and stomata may be species‐specific and have taxonomic value, as in *Amaranthus* genus (El‐Ghamery *et al*. 2017; Terzieva *et al*. 2019). Indeed, stomata density exhibited significant variability and could be a marker of high diversity, even if it only partially discriminates amaranth species. These variables confirm the high diversity in *Amaranthus* morpho‐anatomical traits, even at juvenile stage.

In addition, young leaf hairiness and the significant correlation with stomata density of monoecious species prefigure behaviour in the fully developed leaves. *A. hybridus* and *A. retroflexus* exhibit high hairiness in mature leaves, which affects evapotranspiration, since hairs can create a favourable microenvironment on the leaf surface to maintain an adequate boundary layer to minimize water loss.

Similarly, the microscopic features describing surface roughness‐related traits and the shape of epidermal cells were analysed. This found a pattern in the four species that overlapped without specific significant differences. These traits were major internal factors, in addition to hairiness and stomatal density, that influence leaf wettability, and represent a barrier to several environment stresses and plant diseases (Wang *et al*. 2015). The leaf surface microstructure and its chemical composition are both involved in water adhesion and retention, and are known to vary among species and with leaf age (Nairn *et al*. 2011; Tie *et al*. 2023; Nairn & Forster 2024). Since leaf roughness, together with hairiness traits, influence leaf hydrophobic properties and mechanics of the contact interface, this study contributes to optimal foliar application of agrochemicals in agriculture (Nairn *et al*. 2011). Indeed, an increased adhesion and retention of spray formulation droplets to the leaf surface of crops or weeds influences cost‐effective spray application of formulants and reduces environmental impacts of excess chemical run‐off and pollution (Nairn *et al*. 2011).

Surprisingly, results of the low‐impact multi‐scale phenotyping in the present work proved that leaf phenotypic variability at juvenile stage across weedy amaranths is manly driven by the morphometric traits associated with leaf size and shape, which are often traditionally used as diagnostic of species (Cope *et al*. 2012). At an in‐depth anatomical level, only stomata density and hairiness AR among water‐related leaf traits were analysed. Since these latter traits discriminate between dioecious and monoecious amaranths for leaf micro‐surface, showing glabrous dioecious species, especially *A. tuberculatus*, have lower stomata density and hairiness area, we hypothesize that they could behave differently in *ad hoc* spray‐retention experiments. In particular, the critical surface tension of glabrous amaranths might be relatively low compared to hairy‐leaved species, resulting in lower spray retention with smaller droplets. However, investigations on differences in foliar agrochemical deposition across varieties or species, or on comparison among difficult‐to wet and easy‐to wet plants, considering also different leaf surface characteristics of target plants, is still at an early stage (Yao *et al*. 2014; Papierowska *et al*. 2019; Ji *et al*. 2021; Ma *et al*. 2022).

In contrast, image‐based phenotyping of leaf surface structure at microscopic scale highlighted that these traits at younger stages contribute less to unravelling taxonomic leaf variation across the four species. In contrast, comparison of mature leaves across the four amaranths at later developmental stages revealed evident dissimilarities, not only in micro‐roughness traits, but also in hairiness of the blade (results not shown). Similar conclusions were found for juvenile‐to‐adult phase changes in grasses, where leaf shape was a more reliable proxy than leaf anatomical traits (Sylvester *et al*. 2001). Moreover, whether the pattern of correlations among multiple leaf characters, defined as phenotyping integration (Damián *et al*. 2018), can change or not during ontogeny is still unresolved (Mason & Donovan 2015; Damián *et al*. 2018).

Phenotyping of leaf surface traits, being strongly related to photosynthetic capacity, water conservation strategy, and water/surface interaction, could be used to provide consistent quantitative investigation of traits related to adaptive responses in an ecological context (Pérez‐Harguindeguy *et al*. 2013; Liu *et al*. 2020). The results described here present an innovative opportunity in support of the definition of next generation strategies for sustainable management of the agroecosystems. Moreover, multi‐scale leaf phenotyping analysis could be integrated with further advanced detection techniques, such as remote sensing (Machwitz *et al*. 2021; Janni & Pieruschka 2022). This could be easily extended and adapted for ecophysiological studies concerning amaranth–crop interactions, and also in the context of climate change adaptations.

## AUTHOR CONTRIBUTIONS

All authors conceived the project. AM, SP and SV provided the plant material. FD'E performed the confocal analyses. GE, EP and DS performed the other measurements. GE processed the images to collect the data. FB, MV, DS and EB performed the statistical analyses. DS, EB, EP, FB wrote the manuscript. AM, SP, SV and FD'E revised the manuscript, with the assistance of all co‐authors.

## CONFLICT OF INTEREST

The authors declare no conflict of interest.

## Supporting information


**Figure S1.** Sample image of leaf appearance of the four *Amaranthus* species.

**Figure S2.** Maximum intensity projections of confocal z‐stacks acquired in reflection mode. Samples consist of nail polish imprints of four *Amaranthus* species, obtained from adaxial leaf surface.

**Figure S3.** DIM plot of morpho‐anatomical leaf traits analysed at macroscopic scale. Left panel shows canonical scores of different species calculated considering Can1 only. Right panel describes positive or negative correlation with Can1 of morpho‐anatomical traits, calculated on the basis of canonical scores.

**Figure S4.** DIM plot of morpho‐anatomical traits of leaf surface analysed at microscopic scale. Left panel shows canonical scores of different species calculated considering Can1 only. Right panel describes positive or negative correlation with Can1 of morpho‐anatomical traits, calculated on canonical scores.

**Figure S5.** DIM plot of morpho‐anatomical leaf traits related to evapotranspiration. Left panel shows canonical scores of different species calculated considering Can1 only. Right panel describes positive or negative correlation with Can1 of morpho‐anatomical traits, calculated on canonical scores.

**Figure S6.** Means and LSD response for leaf area trait of four *Amaranthus* species. Bars with different letters indicate significant differences (*P* < 0.05).

**Figure S7.** Mean and LSD response for leaf Dry Weight (DW) trait of four *Amaranthus* species. Bars with different letters indicate significant differences (*P* < 0.05).

**Figure S8.** Means and LSD response for leaf circularity trait of four *Amaranthus* species. Bars with different letters indicate significant differences (*P* < 0.05).

**Figure S9.** Means and LSD response for leaf length trait of four *Amaranthus* species. Bars with different letters indicate significant differences (*P* < 0.05).

**Figure S10.** Means and LSD response for FPO trait of four *Amaranthus* species. Bars with different letters indicate significant differences (*P* < 0.05).

**Figure S11.** Means and LSD response for cell area trait of four *Amaranthus* species. Bars with different letters indicate significant differences (*P* < 0.05).

**Figure S12.** Means and LSD response for cell circularity trait of four *Amaranthus* species. Bars with different letters indicate significant differences (*P* < 0.05).

**Figure S13.** Means and LSD response for hairiness AR trait of four *Amaranthus* species. Bars with different letters indicate significant differences (*P* < 0.05).

**Figure S14.** Means and LSD response for hairiness area trait of four *Amaranthus* species. Bars with different letters indicate significant differences (*P* < 0.05).

**Figure S15.** Means and LSD response for hairiness perimeter trait of four *Amaranthus* species. Bars with different letters indicate significant differences (*P* < 0.05).

**Figure S16.** Means and LSD response for stomata density trait of four *Amaranthus* species. Bars with different letters indicate significant differences (*P* < 0.05).
