# Supplementary figures and images for: Innovative multi‐scale approach to study the phenotypic variation of seedling leaves in four weedy Amaranthus species
Source: Plant Biol (Stuttg). 2024 Dec 11;27(2):310–22. doi: 10.1111/plb.13752 (PMC11846635; doi:10.1111/plb.13752)

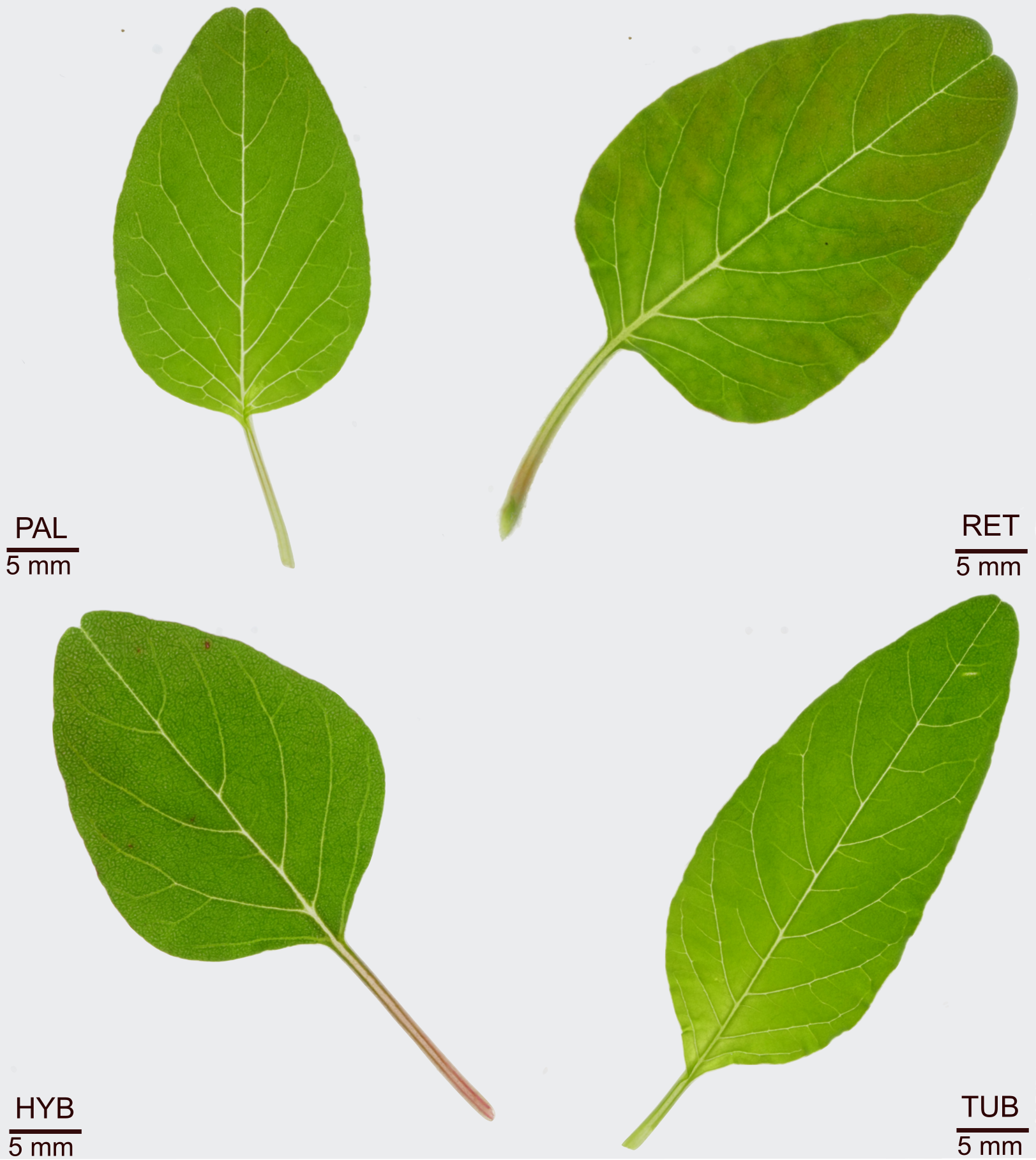

Supplement: Supplementary file 1 — Figure S1. Sample image of leaf appearance of the four Amaranthus species. [file PLB-27-310-s001.tiff]

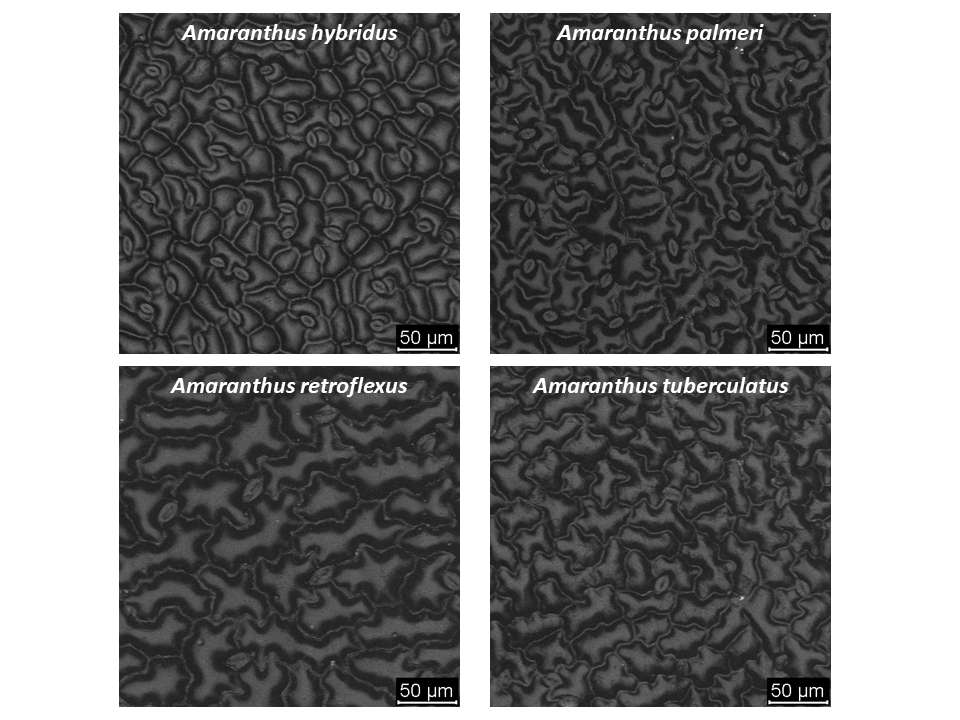

Supplement: Supplementary file 2 — Figure S2. Maximum intensity projections of confocal z‐stacks acquired in reflection mode. Samples consist of nail polish imprints of four Amaranthus species, obtained from adaxial leaf surface. [file PLB-27-310-s005.tiff]

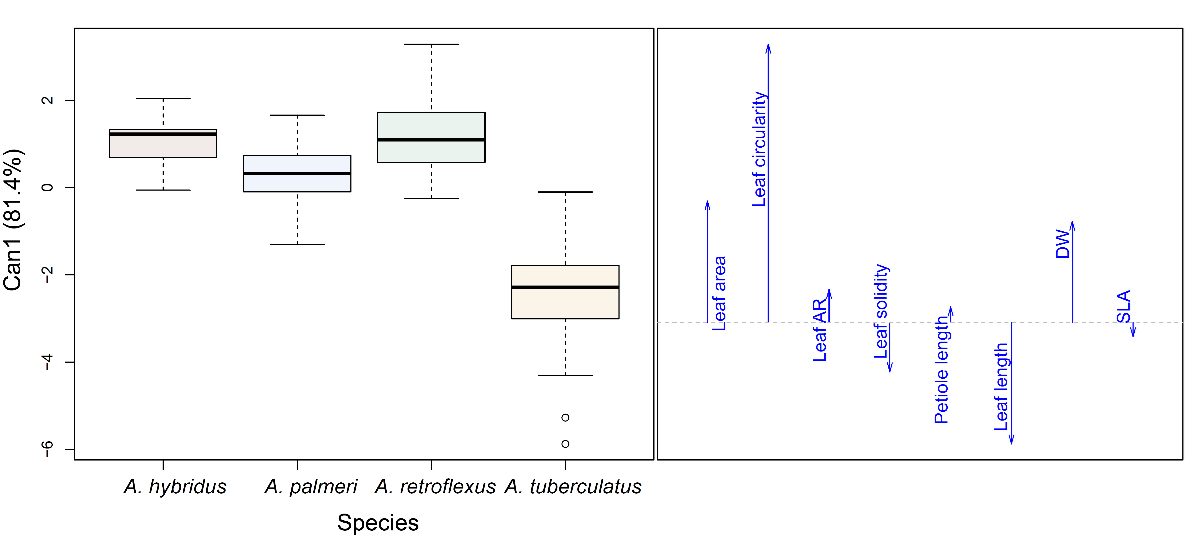

Supplement: Supplementary file 3 — Figure S3. DIM plot of morpho‐anatomical leaf traits analysed at macroscopic scale. Left panel shows canonical scores of different species calculated considering Can1 only. Right panel describes positive or negative correlation with Can1 of morpho‐anatomical traits, calculated on the basis of canonical scores. [file PLB-27-310-s004.tiff]

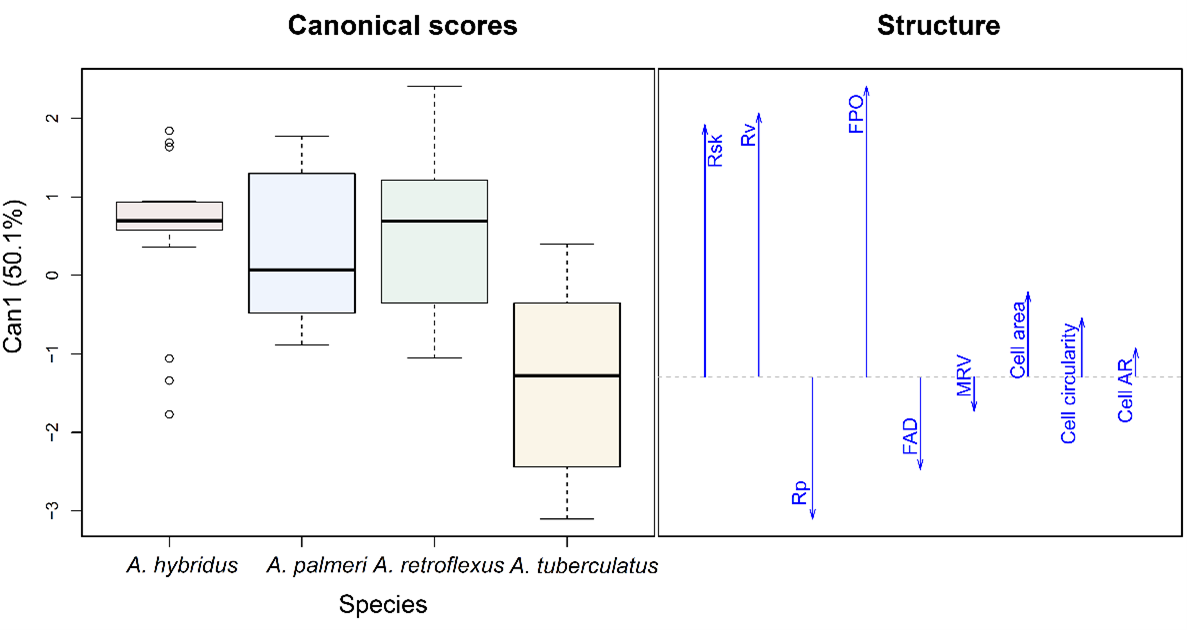

Supplement: Supplementary file 4 — Figure S4. DIM plot of morpho‐anatomical traits of leaf surface analysed at microscopic scale. Left panel shows canonical scores of different species calculated considering Can1 only. Right panel describes positive or negative correlation with Can1 of morpho‐anatomical traits, calculated on canonical scores. [file PLB-27-310-s008.tiff]

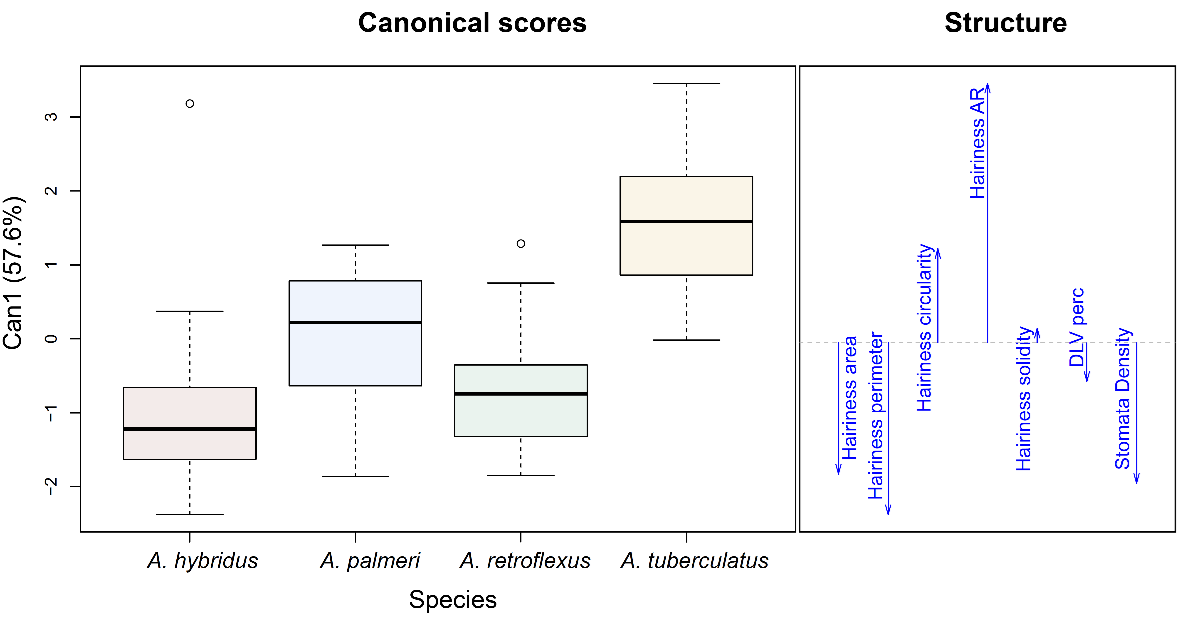

Supplement: Supplementary file 5 — Figure S5. DIM plot of morpho‐anatomical leaf traits related to evapotranspiration. Left panel shows canonical scores of different species calculated considering Can1 only. Right panel describes positive or negative correlation with Can1 of morpho‐anatomical traits, calculated on canonical scores. [file PLB-27-310-s007.tiff]

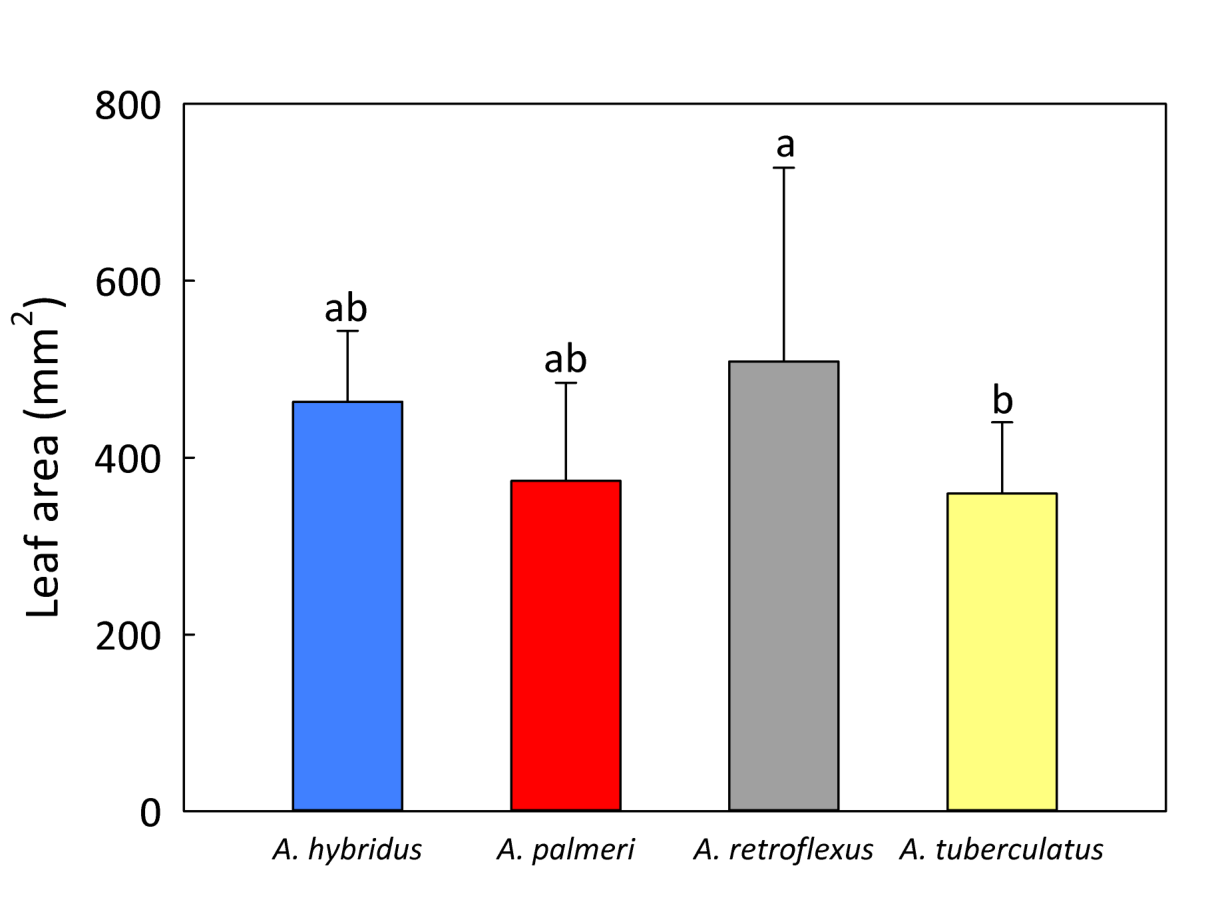

Supplement: Supplementary file 6 — Figure S6. Means and LSD response for leaf area trait of four Amaranthus species. Bars with different letters indicate significant differences (P < 0.05). [file PLB-27-310-s009.tiff]

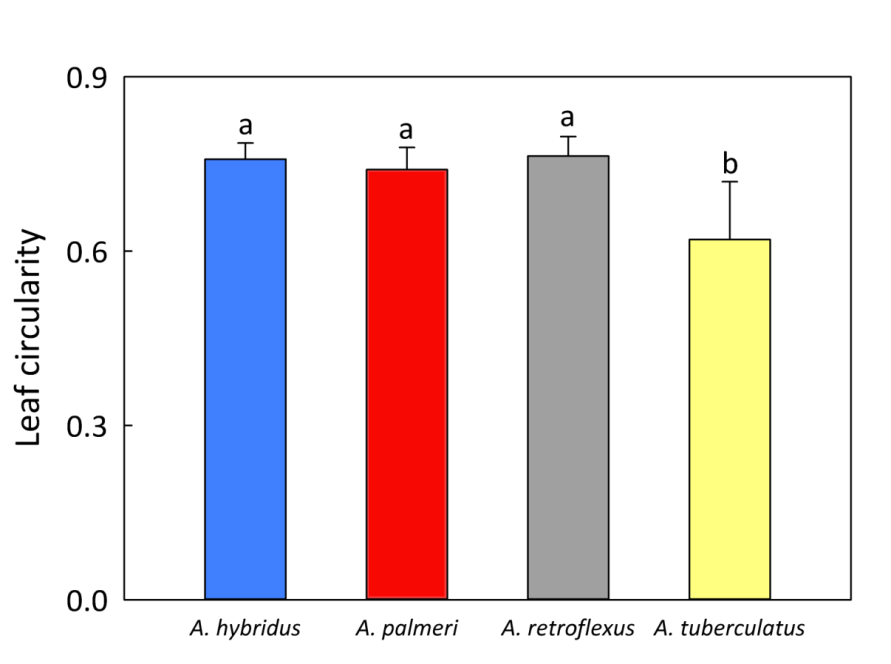

Supplement: Supplementary file 8 — Figure S8. Means and LSD response for leaf circularity trait of four Amaranthus species. Bars with different letters indicate significant differences (P < 0.05). [file PLB-27-310-s011.tiff]

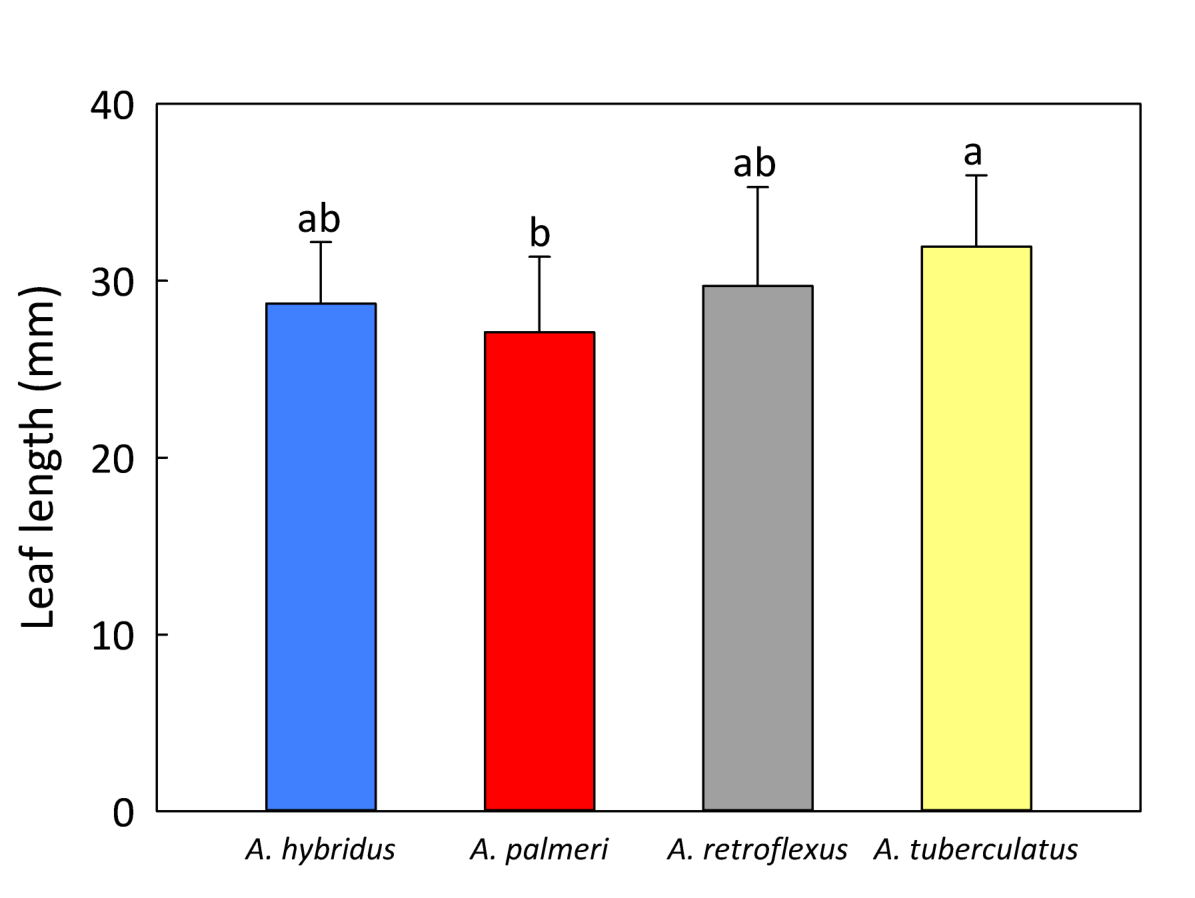

Supplement: Supplementary file 9 — Figure S9. Means and LSD response for leaf length trait of four Amaranthus species. Bars with different letters indicate significant differences (P < 0.05). [file PLB-27-310-s012.tiff]

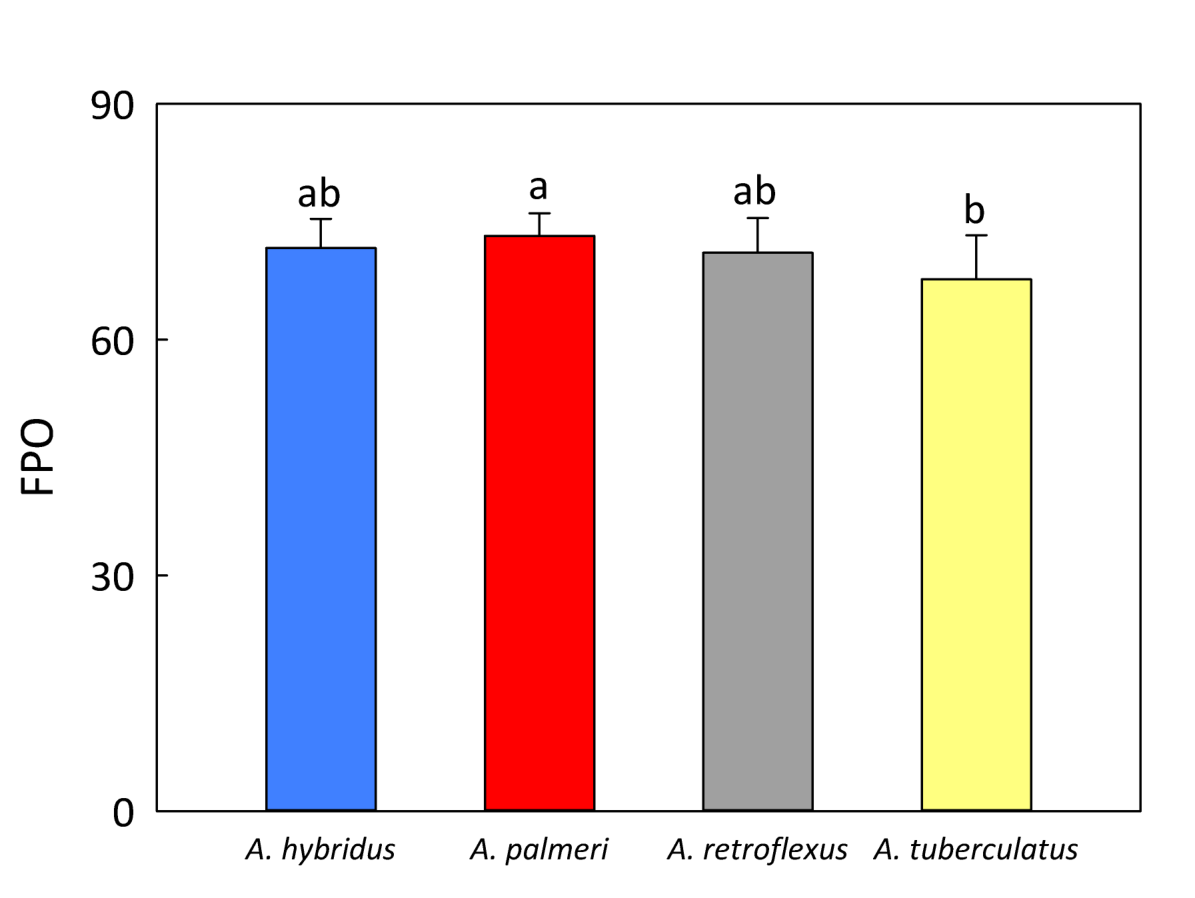

Supplement: Supplementary file 10 — Figure S10. Means and LSD response for FPO trait of four Amaranthus species. Bars with different letters indicate significant differences (P < 0.05). [file PLB-27-310-s016.tiff]

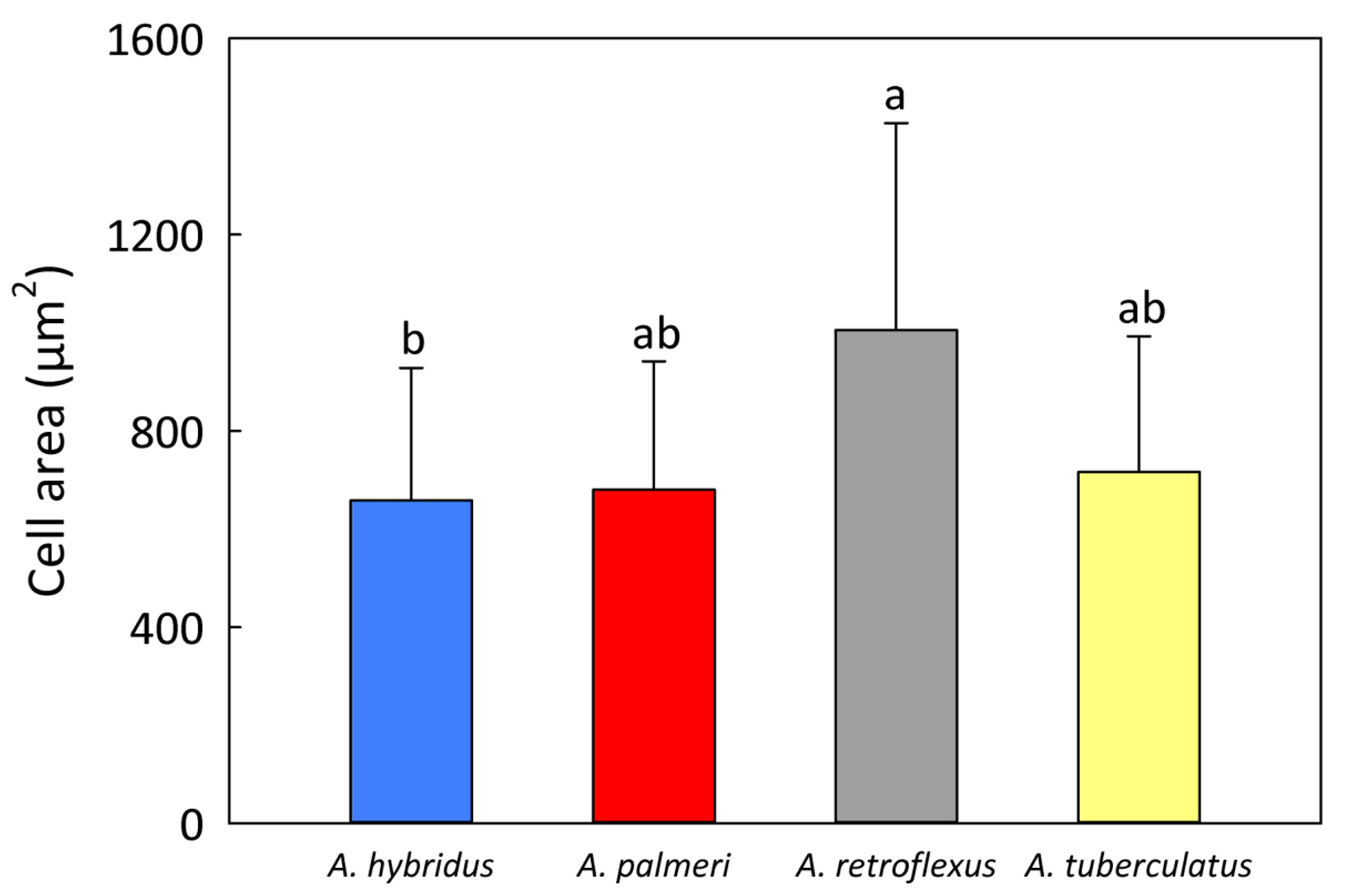

Supplement: Supplementary file 11 — Figure S11. Means and LSD response for cell area trait of four Amaranthus species. Bars with different letters indicate significant differences (P < 0.05). [file PLB-27-310-s006.tiff]

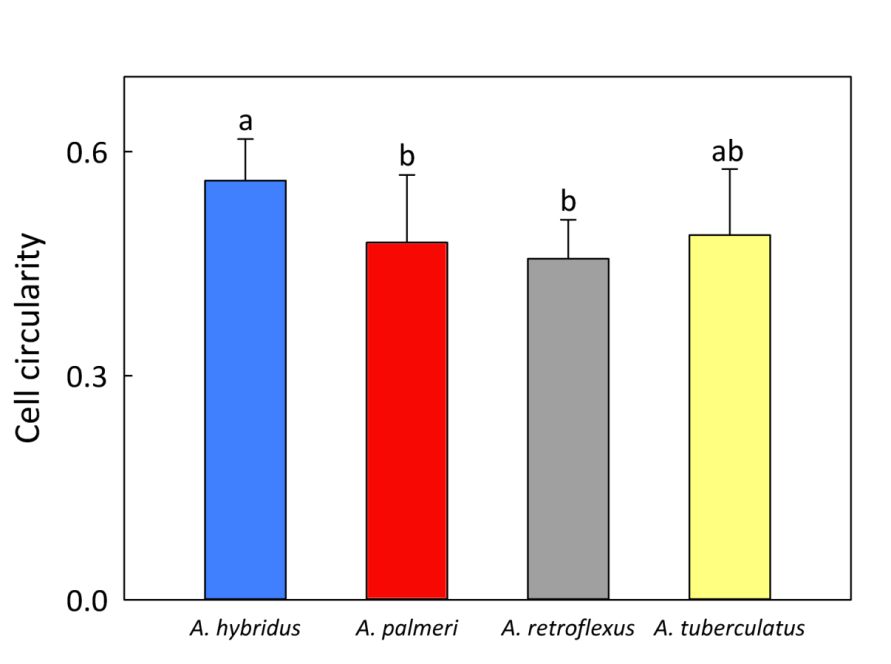

Supplement: Supplementary file 12 — Figure S12. Means and LSD response for cell circularity trait of four Amaranthus species. Bars with different letters indicate significant differences (P < 0.05). [file PLB-27-310-s014.tiff]

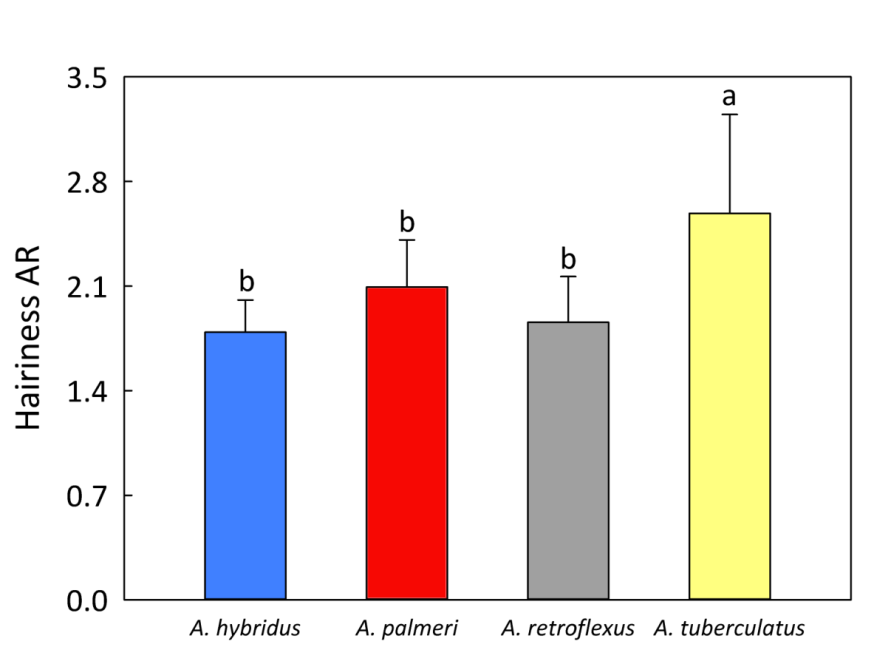

Supplement: Supplementary file 13 — Figure S13. Means and LSD response for hairiness AR trait of four Amaranthus species. Bars with different letters indicate significant differences (P < 0.05). [file PLB-27-310-s015.tiff]

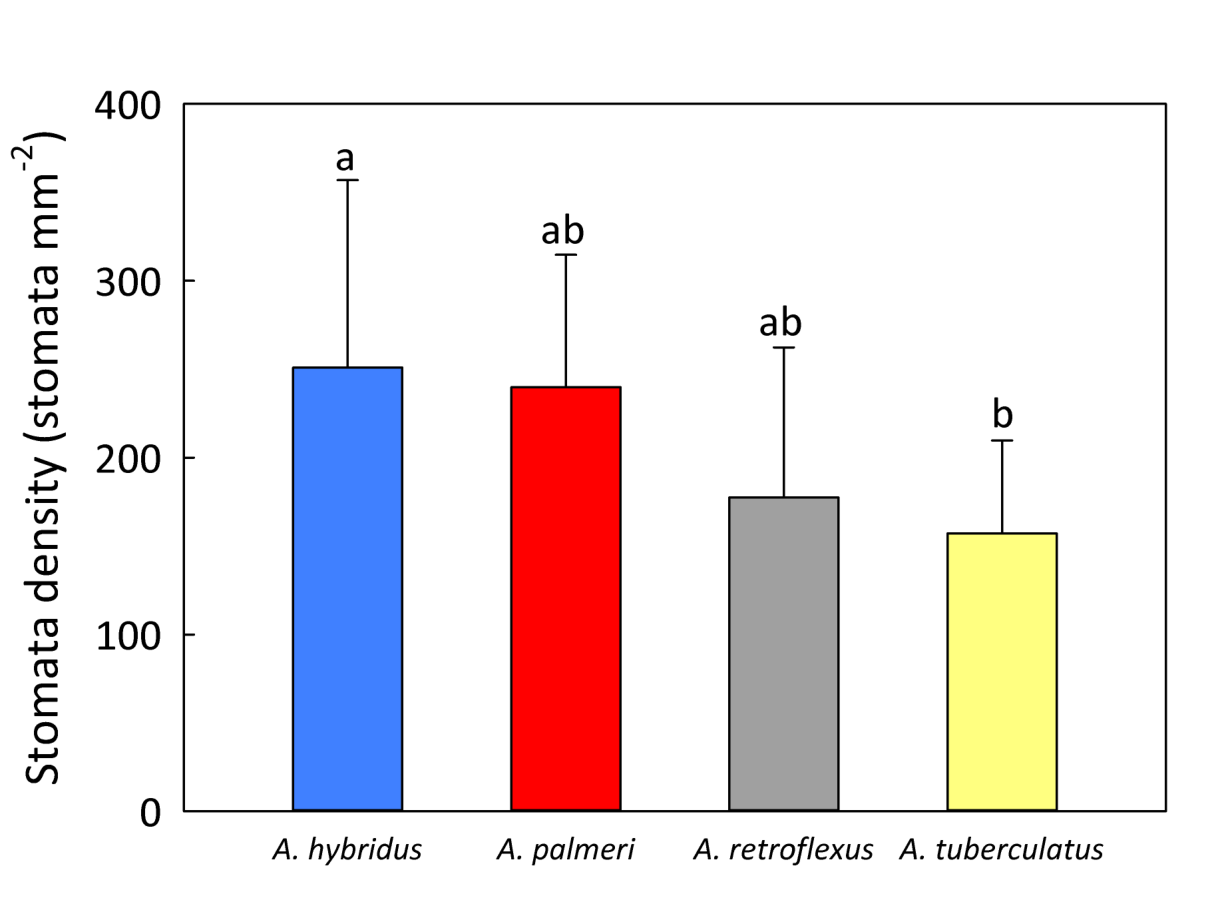

Supplement: Supplementary file 16 — Figure S16. Means and LSD response for stomata density trait of four Amaranthus species. Bars with different letters indicate significant differences (P < 0.05). [file PLB-27-310-s003.tiff]
